# Evaluation of basic surgical skill workshop at undergraduate level in the discipline of surgery

**DOI:** 10.12669/pjms.36.4.1792

**Published:** 2020

**Authors:** Maliha Yunus, Zeeshan Ghani, Ihtasham Muhammad Ch., Ayesha Akram

**Affiliations:** 1 Prof. Dr. Maliha Yunus, MBBS, FCPS, MHPE. Professor of Surgery, Al-Nafees Medical College & Hospital, Isra University, Islamabad Campus, Pakistan; 2 Dr. Zeeshan Ghani, MBBS, FCPS, MCPS. Associate Professor of Paediatrics, Al-Nafees Medical College & Hospital, Isra University, Islamabad Campus, Pakistan; 3 Dr. Ihtasham Muhammad Ch., MBBS, FCPS, FRCS. Associate Professor of Surgery, Al-Nafees Medical College & Hospital, Isra University, Islamabad Campus, Pakistan; 4 Dr. Ayesha Akram, MBBS, FCPS, MHPE. Assistant Professor of Gynaecology, HITEC Institute of Medical Sciences, Taxila, Pakistan

**Keywords:** Evaluation, Basic surgical skill workshop, undergraduate medical students, randomized control trial, Kirkpatrick’s model of evaluation

## Abstract

**Objective:**

To evaluate the effectiveness of basic surgical skill workshop at under graduate level.

**Methods:**

This was randomized controlled study (cross-over design) conducted at Al-Nafees Medical College and hospital from 1^st^ January to November 30^th^ 2017. Undergraduate medical students of Year-5 MBBS were randomized into two groups to undergo surgical skills training. One was workshop or interventional Group-A, other was traditional teaching or control Group-B. Online random sampling calculator was used for randomization. Both groups were given a pretest and post-test in the form of two OSATS station.

**Results:**

Total 49 students were enrolled in the study; Group-A had 25 whereas Group-B had 24 students. There was significant difference (*p*=0.000) in mean post-test scores of Group-A (36.28±6.75) and Group-B (24.17±5.09) out of 53 on OSATS station-1. Significant statistical difference (p=0.000) in the mean score of post-tests of Group-A (26.08±18.34) and Group-B (14.42±9.24) out of 37 was also noted on OSATS station-2. There was no significant difference in mean pretest scores on both stations in both groups.

**Conclusions:**

This study has suggestions in development of curriculum as it provides a quantitative substantiation indicating that workshop teaching as a learning strategy can essentially augment traditional teaching of technical skills to undergraduate medical students.

## INTRODUCTION

In the era of 1950s, Benjamin Bloom with a team of educational psychologists gave the concept of academic learning behaviors.^1^ To assist the designing and assessment of educational learning, he developed a system with different categories of learning behaviors. The result of this effort produced “Bloom’s Taxonomy” which is now a popular entity in field of education.^2^ The goal of this taxonomy is to motivate medical educators to focus in all three domains, (cognitive, psychomotor and affective domains) creating a holistic form of medical education.^3^ In our experience, there has been emphasis on learning of cognition and less attention is paid to the development of learning and teaching strategies of psychomotor domains in medical curricula. The assessment strategies of these skills are also less developed.^4^

Acquisition of psychomotor skills is an important part of surgical training curriculum. Basic psychomotor skills need to be mastered before contemplating more complex tasks as bad habits learned early are difficult to rectify. Psychomotor proficiency is an integral pillar of surgical profession that requires a vigorous program of skills acquisition.^5^ Regarding learning domains, tools have been developed, tested and validated globally as well as locally for knowledge domain in teaching and evaluation of surgeons’ competencies. Psychomotor skills acquisition still relies on the Halstedian model of apprenticeship. In which surgeons “learn by seeing” and “catch if you can” principle. The value of this traditional method of skills acquisition cannot be ignored, however newer learning and assessment strategies of skills attainment at under graduate level need to be developed and evaluated that strengthen the traditional psychomotor skill approach.^6^

In this modern world, everyone is at a higher risk of traumatic injury ever than before.^7^ Anyone having minor or major trauma visits public sector hospitals, where junior doctors are on call. It’s every body’s right and wish to get best possible care and treatment. If our house surgeons who are the primary care givers, treating doctors and back bone of health system not equipped with basic surgical skills, the result of trauma management will be not as good as it is needed.

Mastery of basic surgical skills must be a part of all passing out doctors and now it is strongly recommended that the acquisition of these skills should not be on real patients, as patients are not experimental animals.^8^ Technical performance consists of surgical knowledge, judgment, and dexterity. The focus of this study was to propose a program for teaching the principles of surgery during undergraduate degree program in a medical school.^9^ Success of this study is an advocacy for curricular changes for incorporating these skills in undergraduate curriculum.

Once a skill is inculcated, it is necessary to evaluate it equitably, as the self-reported measures are not reliable.^10^ Hence, a direct measure via an Objective Structured Assessment of Technical Skill (OSATS) can be used to indicate both a baseline level of pedagogical skills and learning which has occurred as a result of a training program.^11^ We conducted structured surgical skill workshop to enhance learning of technical skills of undergraduate medical students and evaluated the effectiveness of basic surgical skill workshop.

## METHODS

This randomized control trial was carried out at Al Nafees Medical College (Ref No. F. 2/IUIC-ANMC/EC/2017, dated September 14, 2017). Two groups were created, Group-A interventional group underwent one-day surgical skill workshop, Group-B traditional teaching group underwent three weeks’ rotation both in operation theater and accident and emergency department. To minimize learning effect of each modality, four weeks’ time lapse was given. A total of 49 students, who fulfilled the inclusion criterion, were enrolled in the study.

### Inclusion Criteria

Willing students of Al Nafees Medical College (ANMC) and Daily Attendance not less than 70%

### Exclusion criteria

Those students who have already attended a workshop from any institute pertaining to surgical skill acquisition may affect the results of study because of their existing knowledge, and were excluded from the study. The total duration of study from inception of the idea to completion was from 1^st^ January to November 30^th^ 2017.

### Standardized OSATS situations (annex ‘I’)

OSATS were based on two daily life scenarios encountered in Accident & Emergency Department or in Operation Theatre.

***Station 1:*** Identification and handling of basic surgical instruments.

***Station 2:*** Instrument knot tying.

**Fig.1:**
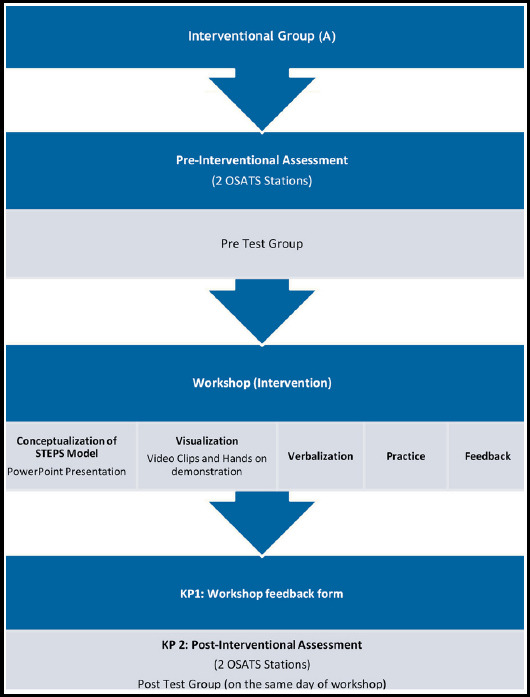
Data Collection Procedure for Group-A.

**Fig.2:**
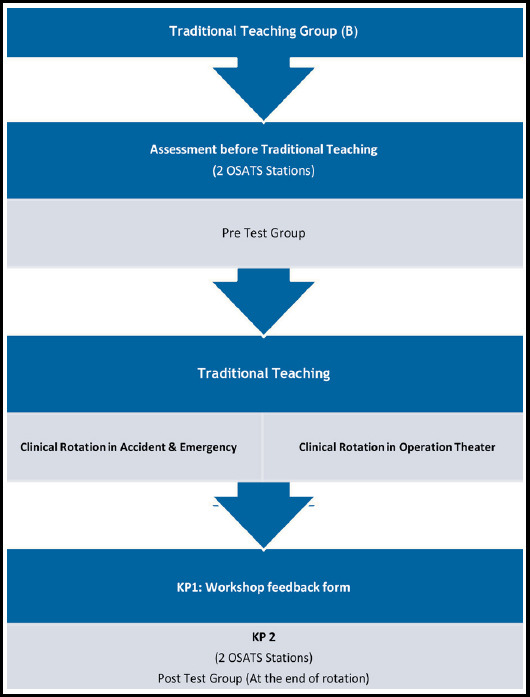
Data Collection Procedure for Group-B.

Data collection procedure is shown in Fig.1 and Fig.2 for Interventional Group-A and Traditional Teaching Group-B, respectively. The purpose of the study was explained to the respondents. Formal consent was obtained on consent form.

The performance of study subjects was assessed on a pre-designed proforma which was filled by the examiners on that specific station. Marks were awarded by all or none principle on dichotomous questions for each parameter and few parameters were assessed by Likert scale method where dexterity of instrument handling and behaviors were assessed. After assessment, cumulative score was calculated for every student and each station, the percentages were calculated and documented. Paired sample t-test was applied for all groups, workshop methodology of teaching (Pre-test, post-test) Traditional methodology of teaching (Pre-test, post-test). All 49 medical students were recruited in the study. There was no drop out in the data collection procedure. Data was entered on excel sheets and then transformed to SPSS version 21. Experimental or workshop group was categorized as Group “A” whereas traditional group was categorized as Group “B”. Experimental and traditional groups were separately compared by applying independent sample t-test. Pre-tests and post-tests means were compared by calculating paired sample t-test of both experimental and traditional teaching group separately on both stations.

## RESULTS

Demographics and baseline information about the participants included their age, gender and their academic placement (year) in MBBS. Forty-nine final year medical students doing clerkship in surgical department were recruited in the study. There was no drop out. All the students were male as we have gender segregation in our classes at Al-Nafees Medical College and all were in the age group of 23-25 years.

Comparison of Group-A, intervention group and B, control group respectively at station-1 by paired sample t-test as shown in Table-I. Pretest and posttest of Group-A and Group-B at station 1 revealed significant difference in terms of statistical analysis. The mean and standard deviation of Group-A in pretest was 23.20±SD and posttest was 36.28±SD out of total score of 53. Degree of freedom (d.f=n-1) degrees of freedom estimate variability. It shows that Group-A performed better in posttest. While mean score of pretest of Group-B was 19.00 and posttest was 24.17, showing better performance of Group-B in posttest as well. P-value of both groups was (p=0.00) at five percent confidence interval, which is showing a statistically significant value between both of the groups included in the study.

**Table-I:** Paired and Independent sample t-test for Group A and B at OSATS 1

Paired sample t-test for Group A and B at OSATS 1						

Groups	n	Mean Score	S.D	t-value	d.f	P-value
Group A						
Pre-test	25	23.20	3.09	-5.93	24	0.000
Post-test	25	36.28	6.75			
Group B						
Pre-test	24	19.00	4.49	-2.742	23	0.000
Post-test	24	24.17	5.09

*Independent sample t-test for Group A and B at OSATS 1*						

*Groups*	*n*	*Mean Score*	*t-value*	*d.f*	*P-value*	
A	25	36.28	4.022	47	0.000	
B	24	24.17				

A significant difference (p=0.000) found between the experimental group (Group-A) statistically by applying Independent sample t-test, which received an intervention Group-A as well as in control Group-B. Group-A secured mean score of (36.28) and Group-B acquired (24.17) which determine that Group-A was comparatively better than Group-B. Group-A and Group-B comparison in terms of Independent t-test implicates a statistically significant difference (Table-I).

Results of station-2 paired sample t-test for Group A and B are shown in Table-II. Pretest and posttest comparison of Group A and B at station-2 showed statistically significant difference (p=0.000). The mean score of pretest of Group-A was 10.48 and posttest was 26.08 out of total score of 37. The mean score of Group-B at pretest was 9.17 and posttest was 14.42. It showed that both groups performed better in posttest. Independent sample t-test on both Groups A & B at station 2 also revealed statistically significant difference (Table-II).

**Table-II:** Paired and Independent sample t-test for Group A and B at OSATS 2

Paired sample t-test for Group A and B at OSATS 2						

Groups	n	Mean Score	S.D	t-value	d.f	P-value
Group A						
Pre-test	25	10.48	6.48	-13.12	24	0.000
Post-test	25	26.08	18.34			
Group B						
Pre-test	24	9.17	6.34	-4.06	23	0.000
Post-test	24	14.42	9.24

*Independent sample t-test for Group A and B at OSATS 2*						

*Groups*	*n*	*Mean Score*	*t-value*	*d.f*	*P-value*	
A	25	26.08	6.11	47	0.000	
B	24	14.42				

## DISCUSSION

Participants were divided into two groups, Group-A was taught with modern workshop methodology of teaching, the intervention group and Group-B was taught with traditional methodology, the control group. Their skills were assessed on two OSATS stations and their performance was documented on a pre-designed proforma based on scoring system. OSATS stations were designed by using low fidelity bench model in dry Skill Lab. Each OSATS station was evaluated prior and after both the teaching methodologies in respective groups. The cumulative sum of each OSATS station was calculated for each groups both pre and post teaching. At OSATS 1station, the pre teaching cumulative sums were approximately the same, Group-A had a mean score of 15 and Group-B secured 12.1. So the basic knowledge of both the groups was same and it holds true for many studies in literature.^12^

But when we compared the post teaching means of both the groups, there was a significant difference between the scores of two types of teaching methodologies. This difference proved significant when we applied independent sample t-test as the p-value was (p=0.000) which is significant at this particular data. It answered the research question of our study in a strong way: Does a structured surgical skill workshop enhance learning of technical skills of under graduate medical students? The aim of research was to prove the effectiveness of surgical skill workshop at undergraduate level. So one can claim that in order to maximize the effectiveness of technical skill development in both under graduate and post graduate medical students, one can apply the same concept of structured teaching, deliberate repeated practice sessions and a constructive timely feedback related to the procedure. An author suggested that Small-group workshops by qualified and knowledgeable tutors are very effective to provide learner-centered teaching.^13^ In a previous study, it was proved that a psychomotor skill gap exists in medical undergraduate training program and Simulation-based workshop are recommended in curriculum of undergraduate medical students.^14^

In contrary to our study’s results, a study showed that when two methods, traditional and psychomotor skill based teaching interactive session for teaching basic nursing skills were compared, no significant differences between the groups’ cognitive gains were detected. However, there were statistically significant differences (*p*=0.01) in satisfaction of students. Interactive group was more satisfied with their teaching approach than the traditional group.^15^

At OSATS 2, the similar trend of cumulative score was observed. Pre teaching scores were almost similar for both the groups. While after teaching, there was a statistically significant difference between the two groups. Again the p value was (p= 0.000) on comparing of both the means. Similar finding was noted in a study whose theme was “Training on a bench model transfers well to the human model”.^16^

Another interesting fact which was noted on Station-2, was that instrument knot tying was performed much better in post- test if we compare Group-A with Group-B scores. On first impression it seemed a researcher’s bias that is “halo effect”. It might be true if we used only “reaction” or first step of Kirkpatrick’s evaluation model but it is eliminated as we used second level of Kirkpatrick’s model that is “learning”. We have done a pre-test post –test analysis to overcome this bias. The reason students were very excited was hands-on activities in this station. They feel empowered and confident while holding surgical instruments. Students might choose surgery as their career because of this useful activity because lesser number of doctors is opting for surgery as their career because of its stress and longer working hours.

Significant difference in pre-test, post-test score of our study is very beneficial for medical educators promoting and sponsoring the concept of skill lab development in all medical schools.^17^ In Pakistan, this concept is getting stronger day by day. We have state of the art skill labs at many places in Khyber Pakhtunkhwa, Sindh and in Punjab as well. Large amount of funding by these institutes towards establishment of skill labs is based on the assumption of better acquisition of both basic and complex procedural skills for both undergraduate and postgraduate medical students^18^ It is extremely important to develop technical skill courses in all surgical specialties and allocate ample time for skill teaching and practice in our curricula.^19^

In this modern era, cost is an important consideration.^20^ Significant difference in the performance of traditional and experimental workshop group has proved that unstructured training of medical students leads to wastage of precious time of students and of already much burdened clinical preceptors as well. They have to plan for monthly rotations of medical students in operating rooms and accident and emergency departments. All this means more number of preceptors, more work stations and breach in the principles of operating rooms. This creates a chaos in accident and emergency department without a substantial gain. Answer to this complex situation is a Well-structured training workshop even with low fidelity models in a skill lab. Model should be instructionally operational. It means that it should be able to teach close to real surgical skills and must be valid in the evaluation of surgical skills as well, so fidelity is relatively less important at undergraduate medical school level.^21^ There must be well planned strategies to expand student experience and capability of technical skills in a supervised and structured manner because their competencies vary widely.^22^

Safety of patients and surgical skill labs are interrelated terms. This holds true after the results of our study. In order to reduce reliance on patients, simulated environment is an appropriate answer.^23^ Now the burden is shifted from operating rooms to the shoulder of course directors. Their responsibility for developing learning outcomes of a course, providing conducive and safe simulated learning environment of skills has increased and much needed in this time frame. Patient’s safety is of paramount importance and it is the pivotal point of whole medical education.

Another very important aspect of this study is long term effects of skill procurement in medical students which will help them in the designing and implementation of future technical skill courses.^24^,^25^ Our study was aimed to teach basic surgical skills in a supervised manner which will give confidence to undergraduate medical students in their future professional life.

### Limitation of the study

The study is carried out in a single center of ISRA University. Further metacentric studies are required to see the effectiveness of surgical skill workshop at under graduate level throughout the country. Further, our study was limited only two surgical skills so we had only two OSATS stations to assess the students. Although, there were two examiners who were independently marking the students in our study but future studies can include more surgical technical skills to teach and assess.

## CONCLUSION

This study has suggestions in development of curriculum as it provides a quantitative substantiation indicating that workshop teaching as a learning strategy can essentially augment traditional teaching of technical skills to undergraduate medical students.

### Recommendation

There is a space for additional inquiry in the field of assessment of usefulness of surgical workshop at undergraduate level. Other important surgical procedures can be added. Cognitive aspect about the possible complications of the procedure and steps to be taken to treat these complications can be part of this extended workshop.

### Authors` Contribution

**MY:** Conceived idea, Data collection, Manuscript writing, Responsible and Accountable for the accuracy or integrity of the work.

**ZG:** Data interpretation, Manuscript writing.

**IMC:** Designed research methodology, Data collection, Manuscript writing.

**AA:** Statistical analysis, Manuscript final reading.
